# Potential Effect of Liposomes and Liposome-Encapsulated Botulinum Toxin and Tacrolimus in the Treatment of Bladder Dysfunction

**DOI:** 10.3390/toxins8030081

**Published:** 2016-03-18

**Authors:** Joseph J. Janicki, Michael B. Chancellor, Jonathan Kaufman, Michele A. Gruber, David D. Chancellor

**Affiliations:** Lipella Pharmaceuticals, Pittsburgh, PA 19019, USA; joe.janicki@lipella.com (J.J.J.); mdato@aol.com (J.K.); michele.anthony@lipella.com (M.A.G.); daviddchancellor@gmail.com (D.D.C.)

**Keywords:** liposome, bladder, interstitial cystitis, overactive bladder, botulinum toxin

## Abstract

Bladder drug delivery via catheter instillation is a widely used treatment for recurrence of superficial bladder cancer. Intravesical instillation of liposomal botulinum toxin has recently shown promise in the treatment of overactive bladder and interstitial cystitis/bladder pain syndrome, and studies of liposomal tacrolimus instillations show promise in the treatment of hemorrhagic cystitis. Liposomes are lipid vesicles composed of phospholipid bilayers surrounding an aqueous core that can encapsulate hydrophilic and hydrophobic drug molecules to be delivered to cells via endocytosis. This review will present new developments on instillations of liposomes and liposome-encapsulated drugs into the urinary bladder for treating lower urinary tract dysfunction.

## 1. Introduction

Instilling drugs directly into the urinary bladder can maximize local drug therapy while minimizing systemic toxicity and side effects [1,2]. Local and topical intravesical treatment is a standard treatment of superficial bladder cancer, and there has been interest in applying intravesical therapy to treat lower urinary tract (LUT) dysfunctions with unmet medical needs [3].

Liposomes are self-assembling lipid vesicles that contain an aqueous core. These can be loaded with drug molecules and facilitate drug delivery across the usually impermeable bladder urothelium. They can also serve to protect the bladder from irritating urine solutes and promote healing of damaged urothelium. Some bladder disorders/LUT dysfunctions that may benefit from liposomal intravesical therapy include:

- Overactive bladder (OAB): A common disorder caused by neurogenic (or idiopathic) detrusor muscle overactivity that results in increased urgency and frequency, nocturia, and urinary pain. A large segment of the patient population has refractory OAB, which is usually not responsive to common oral medications.
- Interstitial Cystitis/Bladder Pain Syndrome (IC/BPS): A chronic inflammatory condition with unknown origin that results in chronic pelvic pain, nocturia, and increased urgency and frequency. Patients generally suffer from debilitating bladder pain. Current treatment options include hyaluronic acid, amitryptaline, pentosan polysulfate, immunosuppressants and topical anesthetics.
- Hemorrhagic Cystitis (HC): A serious condition that results in inflammation and chronic bleeding in the bladder. It can be caused by radiation therapy, chemotherapy, or infection. There are currently no approved treatments, and standard of care therapies, such as hyperbaric oxygen therapy, have a small effect in much of the patient population and can be very expensive. Unless managed properly, HC may progress to a point where a cystectomy is necessary, and can ultimately lead to death.

## 2. The Urothelium

The bladder wall has three well-defined layers consisting of the innermost portion called the mucosa, the intermediate muscularis propria layer (detrusor muscle), and the outer adventitia/serosa layer. The mucosa consists of the urothelium, which has three cell layers: umbrella cells, intermediate cells, and basal cells, and a basement membrane and the underlying lamina propria. The lamina propria contains afferent nerves, interstitial cells, and some smooth muscle cells called muscularis mucosae, which are sometimes absent or not well defined in the human bladder. The mucosa is covered by tight junctional proteins called uroplakins and a glycosaminoglycan (GAG) mucin layer on the luminal surface, which help to provide barrier functionality. The urothelium is a relatively impermeable layer that prevents many urine solutes and drugs from diffusing into the submucosal layer [4]. Furthermore, many drugs have limited stability in the hostile urine environment within the bladder [4,5]. The GAG layer may inhibit substances in the lumen of the bladder from adhering to the urothelium. The tight junctions of the urothelium inhibit the transport of drugs after intravesical administration [6]. Therefore, some drugs or biologic agents that are instilled into the bladder lumen cannot reach desired therapeutic levels in the detrusor wall.

Urothelial damage, such as from IC/BPS, can cause increased permeability that can lead to pain and inflammation. Animal studies have made use of agents that increase bladder permeability, such as protamine sulfate, to simulate the diseased state and to examine drug uptake in the bladder. When treating the bladder via intravesical therapy, it is unreasonable to use harsh agents such as protamine sulfate to facilitate absorption of drugs; instead, drug uptake across the urothelium can be mediated by liposomes in a relatively safe manner. Liposomes can be loaded with many types of drugs [7], but there is growing evidence that empty liposomes can have a significant effect in the urinary bladder. Empty liposomes can help patients with interstitial cystitis by coating and healing the damaged urothelium [8]. Liposomes can carry various drugs, such as botulinum neurotoxin serotype A (BoNT-A), to penetrate urothelium and modulate afferent neurotransmission and potentially treat OAB [2,9].

## 3. Rationale for Intravesical Treatment of Bladder Dysfunction

**Figure 1:**
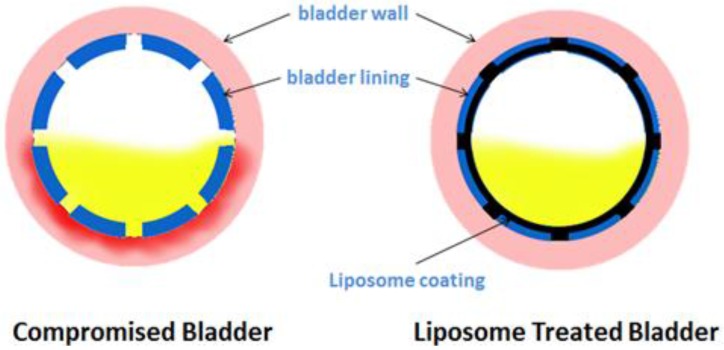
Mechanism of action of intravesical liposome instillations. Liposomes can coat damaged urothelium and protect it from harsh urine solutes.

One of the key reasons for local bladder therapy is to apply an effective dose of a therapeutic drug only to the target organ (Figure 1). The bladder allows easy, safe, and gentle access with prolonged drug exposure [3]. Some of the potential advantages of intravesical treatment include:

- Extending duration of drug contact with urothelium
- Minimizing systemic toxicity side effects
- Repair of urothelium
- Achieving higher drug concentrations in the bladder wall than systemic administration
- Modulating neurotransmission and sensory nerve function

Some patients with a small or sensitive bladder may not be able to hold the drug in their bladder long enough for the drug to work. A reduced drug residence time can decrease therapeutic effects. However, a vast majority of patients can hold a volume of 40–50 mL for 30–60 minutes. Patients receiving bladder instillation therapy should avoid diuretics or excessive fluid intake before and during intravesical therapy to reduce the risk of drug dilution via polyuria.

Liposomes can be used to improve the absorption of intravesically-instilled drugs via endocytosis [7,10,11]. Biophysical studies on interactions of liposomes with cells have suggested that liposomes can be adsorbed, fuse, or transfer lipids with the cell membrane, and that they can be endocytosed [12], making them an ideal drug delivery mechanism. As such, liposomes are the component used for topical application in body cavities such as the vagina and the cul-de-sac of the eye [13,14].

**Figure 2:**
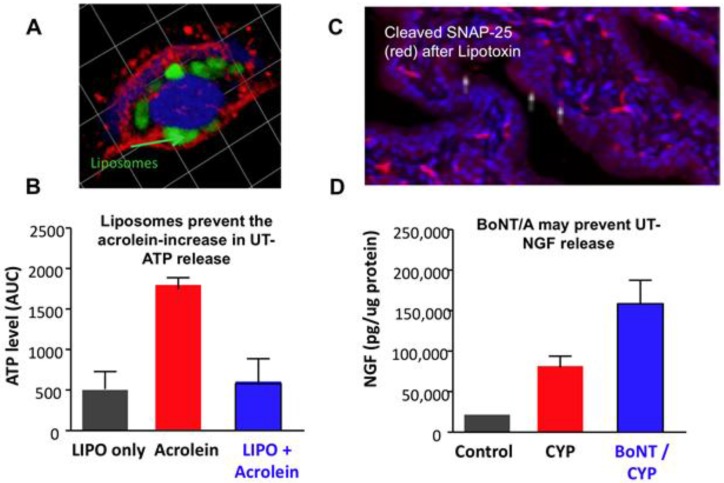
(**A**) Fluorescent labeled (green) liposome within cultured urothelial cell (Phalloidin-red-stains actin filaments; Topro-blue nuclear marker); (**B**) Liposome (LIPO) treatment reduces injury (acrolein) induced ATP release from cultured urothelial cells; (**C**) Urothelial cross section (rat) showing cleaved SNAP-25 (red) following lipotoxin-treatment; (**D**) BoNT-A injection into inflamed (cyclophosphamide (CYP)) rat urinary bladder is associated with increased mucosal-nerve growth factor (NGF) as compared with CYP alone. This suggests that BoNT-A prevents NGF release from urothelium.

Liposome uptake into urothelial cells is likely through an endocytotic process, as supported by experiments demonstrating that liposome-encapsulated gold particles or fluorescently labeled endosomes are taken into the urothelium [15,16]. (Figure 2A). The ability of liposomes to protect cells was studied by inducing toxicity in primary cultured uroepithelial cells with acrolein. Acrolein caused a reduction in cell proliferation and increased ATP release in unprotected cells, while liposome-protected cells prevented injury-induced ATP release. (Figure 2B). Liposomal treatment had no toxic effects on its own. It was shown that liposomes increase the survival of urothelial cells exposed to acrolein and that liposomes bound to the cell surface are internalized via an endocytotic process [17]. The application of BoNT-A was found to result in SNAP-25 cleavage. Figure 2C depicts SNAP-25 cleavage in the urothelium by liposomal BoNT-A (lipo-BoNT) and inhibition of neurotransmitter release. In a cyclophosphamide-inflamed urinary bladder, lipo-BoNT injection increased mucosal-nerve growth factor (NGF) (Figure 2D), suggesting a decrease in released NGF (a marker of epithelium integrity) and thus reduced pro-inflammatory signals. These findings suggest that liposomes can reduce inflammation and repair urothelial barrier function. 

## 4. Clinical Studies of Liposomes for IC/BPS

**Figure 3:**
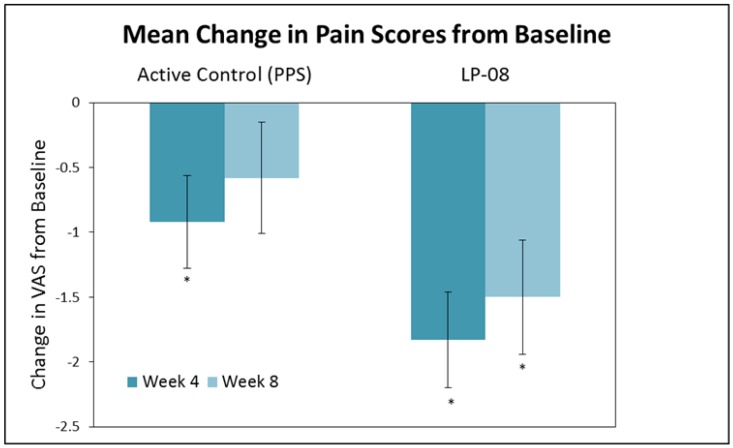
Effect of pentosane polysulfate sodium (PPS) active control *vs.* liposomes (LP-08) on pain caused by IC/BPS. The active control and LP-08 cause a significant decrease in pain at week 4, but the decrease persists only in the LP-08 group at week 8. * Significant difference (*p* < 0.05) *vs.* baseline. Adapted from [8].

Clinical safety and efficacy of liposomes in the urinary bladder was initially reported by Chuang *et al.* [8] in an open-label prospective study of 24 IC/BPS patients. Liposomes (80 mg/40 mL distilled water) were instilled into the bladder once weekly and compared to oral pentosan polysulfate sodium (100 mg) three times per day for a total of four weeks duration. Significant decreases in urinary frequency and nocturia were observed in each treatment group, and statistically significant decreases in the O'Leary-Sant symptom index, pain and urgency were observed in the liposome group (Figure 3). No pain, infection, retention, incontinence, or any other serious side effects were reported by any patients in the study. 

**Figure 4:**
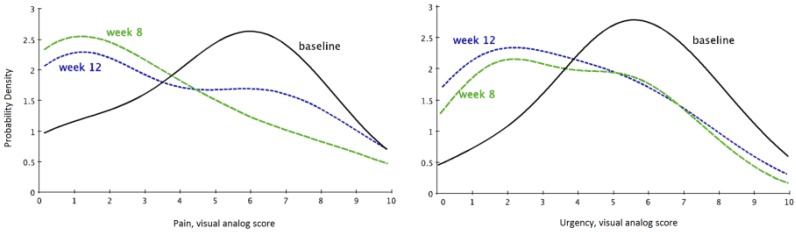
Liposomes instilled into the urinary bladder reduce urgency scores and pain in IC/BPS patients. Probability density functions at baseline, 8 weeks, and 12 weeks are displaced on the two panels. The leftward shift following liposome instillation indicates reduced urgency symptoms and pain. Adapted from [18].

An open-label study in the United States of 14 IC/BPS patients treated with liposomes once a week for four weeks was reported by Peters *et al*. [18]. Pain scores significantly decreased at eight weeks (*p* = 0.01) post-treatment, and urgency scores showed improvement at eight (*p* = 0.076) and 12 weeks (*p* = 0.084) post treatment. The liposomes used in this study (LP-08) were well tolerated and no treatment-related adverse events were reported. Efficacy with LP-08 was associated with improvements in overall symptom score, pain, and urinary urgency (Figure 4).

## 5. Liposomal Delivery of Botulinum Toxin in Treatment of Bladder Dysfunction

**Figure 5:**
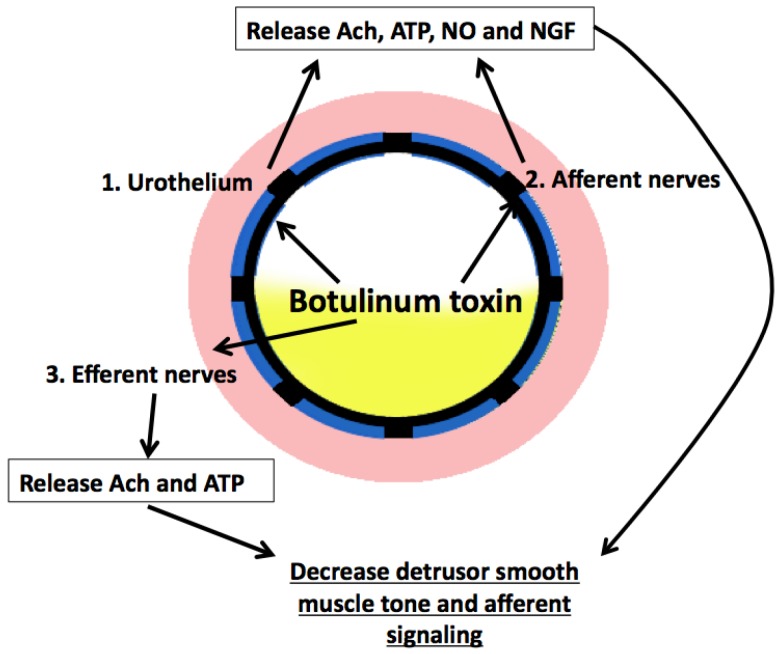
Summary of botulinum toxin effects via intravesical instillation. Potential sites of activity with intravesical instillation of liposomal formulation of botulinum toxin include: 1. Urothelium, 2. Afferent nerves, 3. Efferent nerves.

There are likely multiple effects caused by BoNT-A in the reduction of OAB symptoms, which are summarized in Figure 5. BoNT-A, which has been approved by most international regulatory agencies for idiopathic overactive bladder and neurogenic detrusor overactivity [19], works by inhibiting neurotransmitter release at the presynaptic neuron by cleaving the synaptic vesicle protein responsible for exocytosis of synaptic vesicles [20]. BoNT-A can cause partial paralysis of the detrusor muscle, which leads to an increase in residual urine [21]. BoNT-A can also inhibit afferent neurotransmission by blocking release of sensory neurotransmitters, including substance P and adenosine triphosphate [22]. It has been successfully used via cystoscopic injection in the treatment of overactive bladder and bladder pain syndrome, though this procedure carries risks for bleeding, infection, pain, and urinary retention. Studies showing the depth of lipo-BoNT penetration into the bladder have not yet been completed. Due to the large size of botulinum toxin, it is possible that any mechanism of action from lipo-BoNT could differ slightly from injected BoNT-A due to any restrictions on the transport of the toxin from the urothelium to deeper layers of the bladder. 

BoNT-A is a high molecular weight (150 kDa) neurotoxin that may not be able to gain access to the afferent nerves located immediately below the urothelium without needle injection. Pretreatment of the urothelium with protamine sulfate has been attempted to increase urothelial permeability [23,24,25]. Protamine is a cationic polypeptide that interacts with the anionic GAG layer to increase urothelial permeability [26]. The transport of BoNT-A across the urothelium via liposomes may be increased and BoNT-A encapsulated within liposomes can be protected from degradation by proteases in the urine without compromising efficacy [25]. 

A single center double-blind, randomized, parallel, controlled trial in 24 OAB patients treated with lipo-BoNT was reported by Kuo *et al.* [9]. The subjects in this study were randomly assigned to either normal saline control or lipo-BoNT (80 mg liposomes and 200 U BoNT-A). Primary outcome was the change in urinary frequency as reported on bladder diary at one month post-treatment. The primary outcome significantly improved in the lipo-BoNT group (*n* = 12; *p* = 0.008) but not in the saline group. (*n* = 12; *p* = 0.79). The lipo-BoNT group showed a significant decrease in urgency episodes (*p* = 0.01), and urgency in the saline group did not change (*p* = 0.2). 

**Figure 6:**
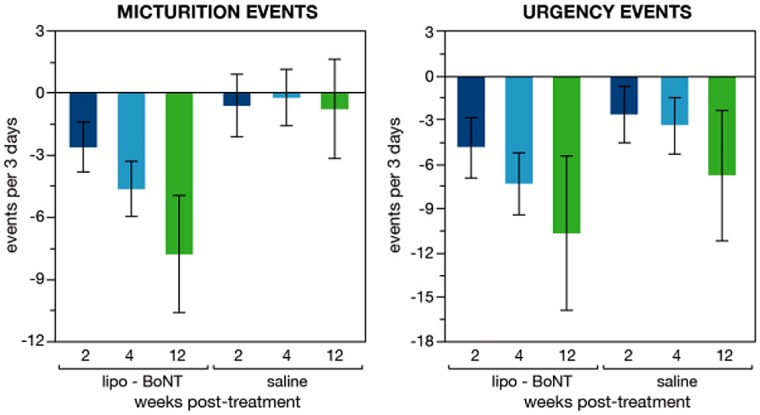
Reduced urinary frequency and urgency associated with lipo-BoNT treatment *vs.* placebo. The treatment-associated frequency reduction (compared to placebo) statistically significant at 4-weeks (*p* < 0.03). Adapted from [27].

Lipo-BoNT was evaluated in a multi-center, double-blind, randomized, placebo (normal saline) controlled study for patients with overactive bladder inadequately managed with antimuscarinics [27]. The study enrolled OAB patients with a mean frequency of >8/day, and either urgency or urgency urinary incontinence (UUI) episodes of >1/day. Lipo-BoNT was administered as a 50 mL instillation solution and was retained for 1 h. The primary endpoint was mean change from baseline in the frequency/day at week 4. Additional endpoints included mean change of urgency and UUI episodes, OAB symptom score (OABSS), and urgency severity score (USS). In total, 55 patients (lipo-BoNT 28, Placebo (saline alone) 27) were analyzed. Lipo-BoNT significantly decreased total frequency to 3/day after four weeks (−4.6 for lipo-BoNT *versus* −0.2 for placebo; *p* = 0.036) (Figure 6). Total urgency (−7.43) and OABSS (−1.86) significantly decreased in lipo-BoNT group at week 4, and no significant difference was observed in the placebo group. USS improved by 39% and 14% for lipo-BoNT and placebo. The results were inconclusive on the effect of treatment on urge incontinence episodes since the sample population had a relatively low baseline incidence. Global response assessment scale improvement was 54% and 32% in lipo-BoNT and placebo group, respectively. Therapeutic effects were observed at week 2 and resolved by week 12. There was no urinary tract infection, urinary retention, or other adverse events in either group. The improvement in frequency and urgency without improvement in incontinence or increasing residual urine volume suggest that the therapeutic effects of lipo-BoNT may be mainly due to blockade of the release of sensory neurotransmitters from urothelium and inhibit afferent activity, but may not have direct effect on the detrusor. 

The Chuang *et al.* study [27] demonstrated that the lipo-BoNT instillation did not cause an increased risk of urinary retention or need for self-catheterization. There is currently an ongoing double-blind, placebo-controlled, multicenter study of lipo-BoNT for IC/BPS listed at https://clinicaltrials.gov/ct2/show/NCT02247557. 

## 6. Liposomal Delivery of Tacrolimus in Treatment of Hemorrhagic and Radiation Cystitis

Tacrolimus is a potent lipophilic immunosuppressive drug. Its mechanism of action involves the inhibition of IL-2-dependent T-cell activation. Topical use of tacrolimus has been shown to help inflammatory skin conditions without systemic side effects [28]. Tacrolimus is essentially insoluble in water, and a liposomal suspension of tacrolimus can increase its efficacy for bladder instillation. In a hemorrhagic cystitis model using cyclophosphamide, liposomal tacrolimus significantly inhibited cyclophosphamide-induced inflammatory cystitis and modulated interleukin, prostaglandin (PG)E_2_, Prostaglandin E receptor 4 (EP) and (IL)-2 function [29]. 

Pharmacokinetics of tacrolimus encapsulated in liposomes (lipo-tacrolimus) was recently evaluated and results demonstrated that the area under the curve of lipo-tacrolimus in the blood at 0 to 24 h was significantly lower than that of tacrolimus instillation or injection [30]. Urine tacrolimus content was significantly greater after intravesical *vs.* intraperitoneal injection of tacrolimus (*p* < 0.05). This study also demonstrated that intravesical liposomal tacrolimus significantly decreased systemic exposure to instilled tacrolimus. 

Liposomal delivery of tacrolimus has demonstrated efficacy in a radiation cystitis rat model [31]. A small animal radiation research platform (SARRP) was used to apply radiation to the rat bladder that was located via CT contrast imaging. It was found that 40 Gy of radiation most reliably produce cystitis symptoms. After 40 Gy radiation via SARRP, there was a significant reduction in intermicturition interval (IMI) values (*p* < 0.05) *vs.* baseline. The animals were treated with either lipo-tacrolimus or saline control four weeks after the radiation. After another four weeks, the mean IMI for the lipo-tacrolimus treatment group returned to baseline levels (*p* > 0.5, baseline *vs.* treatment) while the saline still showed decreased IMI levels (*p* < 0.5, baseline *vs.* placebo). Histology showed that the lipo-tacrolimus treated bladder had minimal inflammation while the saline placebo treated bladder exhibited epithelial changes including swelling and pseudocarcinomatous epithelial hyperplasia.

A case report of intravesical tacrolimus aiding hemorrhagic cystitis (HC) was recently published [32]. An 81 year-old man with a history of prostate cancer was treated with radiation therapy in 2004. There had been no history of prostate cancer recurrence, but the patient developed progressive hemorrhagic cystitis. He was admitted to the hospital twice for nearly 30 days where he required catheterization, bladder irrigation, eight units of blood transfusion for anemia, and two surgeries for fulguration of bleeding. Instead of instillation of formalin as a last resort intervention, the patient received two courses of intravesical tacrolimus without side effects and his gross hematuria resolved and he was discharged after 48 h. His renal function remained stable and he had no further bleeding during the next six months.

## 7. Conclusions

Bladder instillation of liposomes has demonstrated promising efficacy and safety in IC/BPS, OAB, and hemorrhagic cystitis studies. Liposomes improve the delivery of drugs and biologic agents across the urothelium. Liposome encapsulation has been shown to protect drugs such as botulinum toxin and tacrolimus from degradation in the urine. A double-blind, placebo-controlled study of using liposomes with botulinum toxin for IC/BPS is currently ongoing. Intravesical liposome therapy has been shown to be safe in the conducted trials and liposome and liposomal drug delivery may be a promising new therapy for lower urinary tract dysfunctions.

## Abbreviations

The following abbreviations are used in this manuscript:

BoNT-A	botulinum neurotoxin serotype A
GAG	glycosaminoglycan
HC	hemorrhagic cystitis
IC/BPS	interstitial cystitis/bladder pain syndrome
IMI	intermicturition interval
Lipo-BoNT	liposomal botulinum neurotoxin serotype A
LUT	lower urinary tract
OAB	overactive bladder
OABSS	overactive bladder symptom score
NGF	nerve growth factor
RC	radiation cystitis
SARRP	small animal radiation research platform
SNAP-25	synaptosomal-associated protein, 25 kDa
UUI	urgency urinary incontinence
